# Karl Popper Versus Karl Mannheim on Sociology and Democratic Governance

**DOI:** 10.1111/1468-4446.13221

**Published:** 2025-05-05

**Authors:** Martyn Hammersley

**Affiliations:** ^1^ WELS The Open University Milton Keynes UK

**Keywords:** democracy, karl mannheim, karl popper, sociology and policymaking, the public role of sociology

## Abstract

There is a variety of conceptions of the public role that sociology ought to play. Perhaps the most common one presents it as serving a critical or oppositional function, not least in relation to governments and their policies. Yet this has by no means always been the dominant conception of sociology's role. In his well‐known typology, Michael Burawoy recognised ‘professional’ and ‘policy’ versions of the discipline, alongside ‘critical’ and ‘public’ ones. However, even this does not capture the full range of variation in view about sociology's public role. There can be divergencies *within* each of Burawoy's categories. And it is worth taking account of these in order to gain a clear sense of all the possibilities. In this spirit, what is offered here is an examination of contrasting approaches that would fall under Burawoy's heading of policy sociology. These come from two key figures who had considerable influence on twentieth‐century social and political thought—Karl Mannheim and Karl Popper. While they both believed that the function of social science is to serve government policymaking, and both were committed to democracy, they took very different views about sociology's relationship to governance. Indeed, Popper sharply criticised Mannheim's position, condemning it as totalitarian. The issues these authors addressed remain of considerable significance today, and this paper explores what can be learnt from their differences in perspective, as well as from what they shared.

*As a matter of fact, if we believe that we already have the truth, we will lose interest in obtaining those very insights which might lead us to an approximate understanding of the situation. It is precisely our uncertainty which brings us a good deal closer to reality than was possible in former periods which had faith in the absolute.*

(Mannheim 1936, 75)^1^

## 1 Introduction

Both from within and from outside, sociology is often viewed today as taking an oppositional stance towards the status quo; and, as part of this, challenging governmental policies (Turner 1992, 1; Smith 2014). In Burawoy's (2005) influential terms, the emphasis is on ‘critical’ or ‘public’ sociology. But, as he recognised, there has also been a great deal of work under the headings of ‘professional’ and ‘policy’ sociology. And, in addition, he acknowledges some variation *within* these categories. This paper examines differences in orientation that fall under Burawoy's heading of policy sociology—that concerned with directly informing government policymaking.^2^ These differences are illustrated by the approaches adopted by two influential authors in the middle of the twentieth century: Karl Mannheim and Karl Popper. The sharp divergence in their views, along with the problems associated with each position, signal key issues that any notion of policy sociology faces; and some of these issues are relevant to other kinds of sociology as well. They relate to questions about what sociology can supply, what is the function of government and therefore what it needs, and what are the implications of both for democracy (Turner 2014).

In *The Poverty of Historicism* and *The Open Society and its Enemies*, Karl Popper denounced historicism and utopian social engineering (Popper 1944–45, 1945a, 1945b). Among the *contemporary* examples of this set of ideas he cited was the work of Karl Mannheim (Hammersley 2024a). However, while Popper and Mannheim differed fundamentally in their philosophical orientations, both believed that social science should play a central role in democratic governance: they adopted what Popper (1960:III, 20) calls a ‘technological approach to sociology’. Furthermore, while Popper criticises what he sees as Mannheim's commitment to ‘utopian’ social engineering, he himself champions an alternative, ‘piecemeal’, version.^3^

I will begin by outlining Mannheim's conception of the role of sociology in governmental planning, as presented in his book *Man and Society in an Age of Reconstruction* (*MSAR*) (Mannheim 1940).^4^ This was a target of criticism for Popper, and I go on to examine what he says about it, as well as his own ideas about social science and democratic governance. After that, I consider in more detail the very different assumptions on which these two writers rely; and, towards the end, I discuss some of the implications of their work for today.

## 2 Mannheim on Social Science and Planning, and Popper's Criticisms

Mannheim and Popper shared somewhat similar social backgrounds, belonged to broadly the same generation, and even had some commitments in common. Mannheim was born in Budapest in 1893, Popper in Vienna in 1902, both of them into relatively prosperous, assimilated Jewish families whose dominant culture was German. Both suffered the dramatic effects of the First World War, the defeat of Germany and the breakup of the Austro‐Hungarian Empire, as well as the political, social, and economic disorder that these brought about. They watched the establishment of the Soviet Union with interest and also concern, as well as witnessing the rise of the Nazis—from whom they were forced to flee. They were both involved in socialist politics early in their lives, and participated in educational ventures serving the working class. Later, both found exile in England, and taught at the London School of Economics and Political Science, though at different times. Mannheim died in 1947, but Popper went on to have a much longer career, living until 1994.^5^

Reflecting on his experience in Hungary immediately after the First World War, and then in Weimar Germany, and drawing on a wide range of philosophical and social science literature, Karl Mannheim argued in *MSAR* that, if Western liberal democracies were to resist communism and fascism, they would need to adapt to the new social circumstances to which those two political movements were negative responses (Kettler et al. 1984, 81–5; Loader 1985:ch5). He identified the main challenges as the rise of modern capitalism and its consequences—in the form of industrialisation, urbanisation, and greater economic inequalities—as well as the effects of democratisation, of widening political participation to include groups who had no previous direct involvement. Like many others, Mannheim claimed that the first 2 decades of the twentieth century had shown that capitalism must be restrained through government intervention and planning, not least to kerb the poverty and suffering it produced in economic downturns and the political resentment that this generated.^6^ Equally important, he insisted that there must be government effort to socialise the lower classes, as well as new generations, by means of educational interventions designed to counter what he refers to as ‘massification’ (Kettler et al. 1984, 90–1; Loader 1985, 134–5; Yamada 2018). He saw this as necessary to enable these groups both to use the appropriate channels to influence policymaking and to adopt reasonable, not utopian, expectations about what this policymaking could achieve. To some extent, at least, this amounted to their acquiring a sociological perspective on the world, of the kind that he himself was developing: he insisted that sociological knowledge should be central to the curricula of schools and colleges. The aim here was rational control of potentially irrational forces that are creative but which must be directed to serve productive purposes (Floud 1959).

Mannheim argued, then, that government should play a major role in society, by comparison with the much more limited function ascribed to it by liberal theory. What is involved here is not just economic planning but also social reconstruction to facilitate adaptation to new circumstances. And Mannheim saw sociology as providing the knowledge‐base required for this. It offered both detailed empirical information and a theoretical framework which synthesised this to make sense of societal developments. Furthermore, he believed that the process of theoretical synthesis not only provided an understanding of the main social trends and problems relevant to policymaking but also indicated what must be done by government to deal with them (Hammersley 2022b). Here, like Marx (but unlike Max Weber, otherwise an important influence on him), he argues that sociology can provide normative guidance as well as factual information. He writes that: ‘A new type of objectivity in the social sciences is attainable not through the exclusion of evaluations but through the critical awareness and control of them’ (Mannheim 1936, 5). Indeed, he believes that both the possibility and necessity of this is an emergent product of the process of historical development (83–5).

Mannheim was in favour of democratisation—at least in the sense that he regarded it as one of the key trends in modern society that could not be resisted, even less reversed. This reflected his view that our ideals are socio‐culturally constituted, not fixed and inviolate. Nevertheless, his conception of democratic governance involved decision‐making by a political elite, this informed by the work of a cadre of social scientists (Mannheim 1936, 82–3). While members of the political elite were to be elected by citizens, their task was not to carry out the wishes of those who elected them but rather to make decisions in the common interest. Furthermore, Mannheim believed that the process of democratisation—not least the extension of suffrage—must be controlled: that, if it were too rapid, disorder would result, as it had done in Weimar Germany (Mannheim 1936, 106–7).

As noted earlier, Popper fundamentally disagreed with many aspects of Mannheim's views about social science and its role in democratic governance (Popper 1960, 67,75; Popper 1966, 336,351).^7^ He puts forward multiple objections.

1. That Mannheim's ‘planning for freedom’ amounts to ‘utopian social engineering’, and that it is impossible to carry out effectively. This is because social science cannot provide the sort of knowledge of the future required.
2. If too much of society is changed all at once it will be impossible to learn from mistakes, and thereby to improve the situation effectively. For this reason, efforts to transform fundamental social structures are very unlikely to be successful, and will almost certainly have unforeseen consequences, some of which will be undesirable;
3. Attempting utopian engineering leads to tyranny: in other words, it is at odds with the commitment to freedom and democracy that is central to Western civilisation, specifically to the development of ‘open societies’.^8^ Indoctrination and indeed physical violence would be required on the part of the state in any effort to bring about the changes envisaged.
4. Utopian social engineering is associated with the idea that it is legitimate to sacrifice the interests, and even the lives, of some people today in the hope of bringing about a better future. Popper regards this as morally unacceptable, on largely Kantian grounds.
5. Utopian social engineering assumes that perfection could be achieved, when all experience of social life indicates that there can, at best, only be *improvements* in current conditions. This is partly because there are multiple, conflicting conceptions of the good life: Popper insists that, at most, there is only likely to be general agreement about what is wrong, in particular about the need to reduce suffering (Popper 1960, 75).

Despite these differences in view, as I have already indicated, Mannheim and Popper shared a view of sociology as playing a key role in ‘social engineering’. In other words, they believed that it fulfilled a ‘technological’ function (Popper 1960, 58–63).

## 3 Technological Social Science

The idea of sociology as ‘technological’—in other words, that it should be designed to provide guidance for government policymaking in dealing with social and political problems—is only one of several conceptions of its role that can be found in its history, though it has been highly influential. As I noted at the start, it is often referred to today as policy sociology. In crude terms, this view can be contrasted, on the one hand, with a more academic conception, according to which research contributes to a cumulative body of knowledge that is of value in itself (this albeit often accompanied by the hope that it will also have indirect practical benefits), and, on the other, with the idea that research ought to be directed exclusively towards serving ‘progressive’, oppositional forces within society (Rule 1978; Hammersley 2000). These broadly correspond to what Burawoy (2005) referred to as ‘professional sociology’, on the one hand, and ‘critical’ or ‘public’ sociology, on the other.

While there was agreement between Mannheim and Popper about the ‘technological’ role of social science, they differed sharply over exactly how this role should be performed. As I explained earlier, for Mannheim sociology produces empirical evidence, synthesised by a theoretical perspective that supplies both diagnoses and recommendations for the treatment of societal problems through government planning. However, he gave little attention to the character of empirical research and its products, largely taking the validity of these for granted to the extent that they fitted what he took to be the emerging synthesis. This notion of theoretical synthesis seems to derive from his argument in *Ideology and Utopia* that a rational theoretical framework can be developed through the sociology of knowledge (Mannheim 1936; see Kettler 1975; Kettler et al. 1990; Hammersley 2022b). This requires social scientists to engage critically with the various competing political ideologies prevalent in the contemporary situation, with a view to identifying the core of truth in all of them, and the implications of this for practice.^9^

The sociology of knowledge requires understanding not just the ‘internal logic’ of each ideology but also its relation to the social positions of those who promote and adhere to it. This is not so much a matter of individual biography but of identifying the social issues to which an ideology is a response, which social categories find these pressing matters, and what the ideology seeks to secure or change. The only extended example of such analysis Mannheim provides is his study of conservatism in nineteenth‐century Germany (Mannheim 1953), though he offers briefer discussions of this and other ideologies in *Ideology and Utopia* (Mannheim 1936). His argument here blends a Hegelian mode of thought with the sorts of historicist and hermeneutic philosophy that were widespread in nineteenth‐century and early twentieth‐century Germany, plus a Marxist emphasis on the social grounding of all ideas, and on the need for empirical sociological investigation.

By contrast, Popper's prime methodological model is natural science, whose method is hypothesis‐testing—indeed, seeking to falsify theories—with those theories that survive this process forming part of scientific knowledge until further notice. He argues that hypotheses cannot be *proven* true, only shown to be false with any certainty—this is his fallibilism. In its strictest form, such inquiry involves experimental method, which allows the control of potentially confounding variables. However, Popper recognises that this method cannot be used in historical investigations or for that matter in much of social science, but he still regards the testing of hypotheses against evidence as central here too, even if it has to be carried out by other means. Indeed, he argues that ethical and political ideas can be rationally appraised through discussions between parties with very different views, so long as participants are all committed to learning from the process (rather than simply ‘winning the argument’) (Popper 1966:Addendum 1). As I have noted elsewhere, here he comes quite close to Mannheim's notion of dialectic, albeit without the Hegelian undercarriage (Hammersley 2024a).

There are a couple of complications to Popper's picture of the unity of method across the natural and social sciences. First, he draws a distinction between theoretical and historical sciences, the former are concerned with testing general causal claims, the latter with explaining particular events (see, for instance, Popper 1960, 143–7). This does not correspond to the natural/social science divide, in that Popper insists that there are theoretical social sciences. However, in the case of history he suggests that many of the theories employed to produce explanations of events are not in need of, and perhaps cannot be, tested. At the same time, it seems that Popper did believe that sociology was a social science that could test general ideas to discover what were likely to be unintended consequences of particular lines of action. But the examples he uses are generally economic ones. Indeed, to a large extent, his conception of theoretical social science seems to be modelled on Austrian economics, probably at least partly under the influence of Hayek. Unfortunately, he does not address the issue that was a major dispute among the Austrians: whether the discipline's theoretical principles, such as the ‘laws of supply and demand’, are true a priori, and perhaps even analytic in character, or whether they are a product of empirical testing.^10^ Furthermore, it is not clear how his notion of a theoretical sociology would fit with his insistence that social science must be ‘technological’ in character, concerned with solving practical problems.

A second complication is that Popper argues that a distinctive feature of social science is the use of situational analysis, in which there is an attempt to understand people's behaviour as a rational response to the situations they face, with a view to explaining this behaviour and identifying potential unintended (and perhaps also unforeseen) consequences (Popper 1960:Section 31). He may have derived this from Hayek's (1943) notion of a ‘pure logic of choice’, but (in the manner of Weber) he treats it as combining both the interpretation of meaning and causal analysis. Popper draws a parallel between the role that the rationality principle plays in explanations of human behaviour and the ‘animating’ function that some natural scientific laws serve in deductive‐nomological explanations of physical events (but see Stokes 1998, 84–6).^11^

## 4 An Assessment: Sociology's Role in Social Planning and Social Engineering

Neither Mannheim's nor Popper's conception of the nature and role of sociology is beyond criticism, of course. Popper may well be correct that social science cannot play the grand role that Mannheim assigned it. The problem here arises, in part, from the range and amount of knowledge that would be required by a government if it were to engage in the scale of planning that Mannheim envisages. The needed knowledge was not available at the time he was writing, nor (arguably) is it available today (Hammersley 2015). There are also questions about how knowledge from different social sciences is to be synthesised in such a way as to produce the overarching perspective that Mannheim believes is required. This sort of synthesis is more complex than that involved in meta‐analysis, or the various forms of qualitative synthesis that have been developed more recently (Hammersley 2013:ch11). This is because the aim is a comprehensive theory of the whole society, its past development and likely future trajectory.^12^ A further problem is that Mannheim assumes that this process of theoretical synthesis can provide normative guidance about what are the most important problems and how they should be dealt with.^13^ In my view, Popper is right to deny that this is possible, and to point out that it overlooks the fundamental conflicts in value commitments and interests to be found within any large‐scale society.

The alternative to Mannheim's ‘social planning’ that Popper recommends—‘piecemeal social engineering’—involves a focus on resolving specific problems whose importance is generally agreed by citizens. This requires considering possible solutions, their likely success and costs; trying out one or more of these; and monitoring the results. Here, the aim is to alter just those particular aspects of society that are relevant to tackling a problem, and/or perhaps initially applying the changes in specific localities before extending them across society more generally; though Popper insists that piecemeal social engineering does not rule out major governmental intervention in the economy or in civil society. As I highlighted, the distinctive feature for Popper is that piecemeal modification of institutional arrangements allows learning from errors, and thereby improves the chances of bringing about social improvements effectively, and without unacceptable costs.

Popper (2008, 172) acknowledges that ‘piecemeal social engineering’ is not the most appealing of labels, but insists that what it refers to is more feasible than its utopian alternative; and its demands on social science are certainly much less than those of Mannheim's ‘planned society’. It requires social scientists to provide information about particular problems, the sources of these, and the likely success of various policy options in dealing with them, rather than a comprehensive understanding of the whole society and its expected direction of travel. In these terms, piecemeal social engineering may be a more appropriate companion for social science; however appealing its utopian counterpart, or Mannheim's social planning, might be for many of us.

At the same time, the very idea of unintended consequences, which is given great emphasis by Popper, indicates that there are systemic relations among different parts of society, and much sociology (of diverse kinds) has been precisely concerned with these. For example, this was a central feature of structural functionalism (Giddens 1977), of later forms of system theory (Buckley 1967; Luhmann 2012), and of most forms of Marxism (see, for instance, Cohen 1978; Van Parijs 1981). This suggests that a more holistic theoretical approach than he allows may be required if the necessary understanding of particular social problems is to be achieved.

Furthermore, it remains unclear just what relationship Popper envisaged between social science and piecemeal social engineering. In much of his discussion he appears to equate them; at least in the case of social science's applied, rather than theoretical, aspect.^14^ One form that Popper's technological social science could take is that which has come to be associated with advocacy of evidence‐based policymaking and practice. This is in line with what Campbell (1991) called ‘the experimenting society’. Here, prior investigations are carried out into the effects of particular policies or practices, ideally employing randomised controlled trials (RCTs). These are treated as providing knowledge about the relative effectiveness of different policies, on which policymakers can rely in making decisions (but see Deaton and Cartwright 2018; Hammersley 2013:ch4).

However, it is not certain that this approach is in line with Popper's position, since it preserves a distinction between hypothesis‐testing and the application of knowledge that his notion of a technological social science appears to deny. An alternative that may be a closer match is action research, for instance of the kind championed by Kurt Lewin (1946; see Marrow 1969).^15^ However, this does not offer even the control over variables supplied by RCTs, which might be required for testing the effectiveness of an intervention in Popper's terms. Furthermore, in practice, this type of action research has usually revealed a sharp tension between its research and action components; in more abstract terms, between scientific and practical rationality.^16^

There is also a problem with Popper's notion of technological social science that stems from the parallel he draws with natural scientific investigation. This relates to his core claims about scientific method. He insists that knowledge consists of conjectures that have withstood attempts to falsify them, but which may themselves fail the test in the future. This near‐sceptical fallibilism raises questions about whether the knowledge produced by the kind of social scientific investigation Popper recommends could supply what is required even for piecemeal social engineering: it does not offer a body of proven knowledge whose validity can be relied upon, as against the opinions of lay people.^17^ And, strictly speaking, Popper's rejection of inductive argument could suggest that what is currently treated as knowledge is no more likely to be true than the wildest hypotheses. Furthermore this applies not just to high level theories but even to the evidence against which these are to be tested—it, too, necessarily relies upon theoretical assumptions, as Popper recognises. This is not a very solid basis on which to recommend policies that will have major costs and significant consequences for people's lives. Perhaps even more problematic is that it is a weak rationale for *justifying* policies in the public sphere, especially to those who will be affected by them, and those who disagree with them. The sort of fallibilism Popper adopts would appear to recommend very limited governmental intervention, if this is to be based on scientific evidence.^18^

A more general problem with Popper's model of science is also relevant here: that he provides little or no epistemic basis for the rational pre‐selection of hypotheses to test or of policy interventions to try. He seems to imply that any hypothesis must be treated as having the same likely validity as any other before it has been subjected to test. Yet, not all potential hypotheses or possible interventions can or should be trialled (Rescher 1978, 53–7). Furthermore, whereas Popper's suggestion is that, in science, the preference ought to be for the boldest hypotheses, piecemeal social engineering appears to involve precisely the opposite approach.^19^ In the context of policymaking, there are often many interventions that could be made; and these will vary in their likely costs and risks, as well as in how these are socially distributed, so that which interventions are to be trialled should not be, and will not be, a matter of indifference. Furthermore, while the costs of piecemeal social engineering may not be as great as those involved in utopian social engineering, they are still likely to be substantial, given the need for repeated testing of potential solutions. Also, where Popper assumes that citizens will evaluate the effects of interventions objectively, in terms of whether they improve the situation, their evaluations are likely to rely on conflicting values and interests to which they have strong attachments.

So, there are some challenging questions about the viability of Popper's conception of a technological social science, just as there are about that of Mannheim. It also seems to me that both views imply that much of what has been done, and is still done, under the heading of social science is misconceived or worthless—since it is not directly geared to serving policymaking. Equally important, in the case of both writers, the development and implementation of policies appears to be viewed as primarily a technical matter. There is little recognition of the sorts of conflict, bargaining, compromise, manipulation, and struggle that normally come under the heading of ‘politics’. This is perhaps especially surprising in the case of Mannheim.

## 5 An Assessment: Democratic Governance

Both Mannheim and Popper were committed to democracy, albeit in ways that did not necessarily treat it as of value in itself. At the same time, they held very different conceptions of its character. Mannheim defines ‘democracy’ as guided by the principle that ‘all social classes shall be politically active’ (Mannheim 1940, 72). So, for the most part, he treats it as a matter of the breadth of citizenship—of the degree to which the voices of those previously excluded from the political process are now heard within it. And he regards extension of the citizen body as an intrinsic feature of the development of modern societies. This was an influential understanding of ‘democracy’ in the 19th and early 20th centuries, and one that is still widely adopted today.

By contrast, for Popper democracy is the opposite of tyranny: its primary feature is that governments can be changed through elections, so that violence is not required to achieve this. To a large extent, this reflects a liberal perspective in which the main function of government is to preserve the freedom of citizens to live by their own lights. Elections provide an opportunity to counter any illegitimate infringement on that freedom. Popper rejects the idea that the state should impose particular values, or a particular way of life, on its population. This attitude presumably also stems from his belief that there is no rational means of determining conclusively what is the good life, even though rational discussion of this is not ruled out. And, among liberals, democracy is also viewed, often, as a pragmatic adaptation to the fact that there is likely to be sharp disagreement within any population about the good life, and about how society should be organised in order to allow its pursuit (see, for instance, Gutting 1999). However, for Popper, probably the most important reason why freedom must be preserved is that it allows space for the public criticism that is required if rational problem‐solving is to take place. This problem‐solving is necessary both for building knowledge and for bringing about improvements in people's lives. Democracy facilitates this.^20^

At the same time, Popper's position goes beyond liberalism of this kind, in a way which brings him somewhat closer to Mannheim's socialism: he qualifies the principle of freedom not just in the usual liberal fashion—that one person's exercise of freedom must not impinge unnecessarily upon that of another—but also through what has been called his negative utilitarianism. He argues that, while there will be little agreement among citizens about the good life, there is likely to be much more agreement about the need for governments to tackle unnecessary suffering: a democratic majority should identify this, and it is the task of government, aided by social science, to remedy it. We should note that this will inevitably restrict the freedoms of some people, over and above what would be justified in terms of pure liberalism (Shearmur 1996). Popper's negative utilitarianism reflects his early commitment to socialism, and a desire to find a third way between it and liberalism (Hacohen 2000; Popper 2008:ch13).

Even so, Popper's overall stance is closer to liberalism than to socialism, because his primary emphasis is on the need to preserve freedom, so as to allow the sort of public criticism which he believes is the only way of reliably producing knowledge and bringing about social improvements. Prospective policies and their implementation are subject to criticism in the public sphere that can lead to their modification or even abandonment; and this is also where sociology comes in. However, he has a very particular understanding of the form that this discussion should take: it must be guided by tolerance of different views, and by a shared commitment to discovering improved solutions to problems. His model here is how he believes scientific communities operate, or ought to operate.

On the basis of this conception of democratic governance, Popper argued that Mannheim's ‘planned society’ threatens to become a tyranny, in the sense that it demands central control.^21^ In particular, he objected to Mannheim's idea that citizens must be ‘educated’ into accepting the principles of the new form of society if the problems created by capitalism and democratisation are to be dealt with effectively. Popper believed that this amounted to indoctrination; it created dogmatism, undercutting the possibility of critical discussion which, as we have seen, was for him the engine of social improvement. He comments: ‘this, clearly, removes any possibility of testing the success or the failure of the new society. For those who do not like living in it only admit thereby that they are not yet fit to live in it; that their human impulses [need further] organizing’ (Popper 1960, 70). Popper believed that Mannheim's planned society would also necessarily involve violent repression, as was evident in the Soviet Union and the Eastern European countries under its control. So, the planning Mannheim recommended would not produce the results intended, but it *would* generate many unintended and undesirable consequences; and these could prompt further disorder and repression.

Popper may well be correct to warn about the dangers of Mannheim's conception of the planned society, but his alternative is also vulnerable to criticism: not only is his ‘open society’ at odds with how existing Western democracies actually operate, but it is also incompatible, I suspect, with any feasible reform of them. One issue here is that Popper assigns a rather passive role to governments in identifying problems: as we have seen, they are to tackle those identified by citizens, and to do this only in a way that is acceptable to them.^22^ Yet there are reasons why governments must play a more active role, not least in responding to major health emergencies or crises in international relations, even in the face of public opposition. Furthermore, Popper seems to assume that the attitudes and behaviour of citizens will generally conform to his view of what rationality involves. Yet, the level of political participation in Western democracies, even in terms of voting, has long been low, raising questions about the democratic ideal of active citizenship.^23^ Also, the form that public discussion of policies actually takes is frequently a very long way from what Popper's concept of ‘the open society’ assumes: it is often dogmatic, relying on manipulative rhetorical strategies, and even threats of violence.^24^ Any attempt to remedy this would surely involve education and probably repression of those unwilling to comply with the requirements. For these reasons, Popper's conception of the open society may be utopian (Shearmur 1996, 122), and Mannheim's view more realistic.^25^

It could be argued that this problem is even worse today than in the past. For example, the distinction between factual reports and opinion pieces has become increasingly blurred in news media; and some of these, as well as some politicians, invent ‘facts’, frequently appealing to and/or promoting prejudices among their audiences. Meanwhile, on social media, fake information and conspiracy theories, as well as bogus and manipulative argument, are used by advertisers and by ‘influencers’ of many kinds, some funded by companies or by foreign states seeking to shape public opinion. I suggest that, in light of this, Mannheim's warnings that citizens must be socialised into democratic participation remain pertinent; while Popper's explicit rejection of the idea of authority, even of scientific authority, works against this—indeed, it may undercut the prospect of the sort of rational policymaking he championed.

There are also genuine questions about the truth of Popper's assumption that the aggregated views of citizens will correctly represent their individual interests; and even about his belief that people know what is in their own best interests, when it comes to public policy. There are doubts about the competence of many citizens, even that of the most educated, to make sound judgements about policy issues, whether about levels of taxation, government interventions in the economy, diplomatic relations or decisions about war and peace.^26^ This would suggest, as Mannheim insists, that there is a substantial role for expertise, both scientific and practical, and that this must be treated as authoritative (though not accepted on blind faith) if it is to be effective. In short, there are tensions between democracy and expertise that Popper's position highlights, but to which he gives little attention (see Hammersley 2022a).

Finally, I suggest that neither Mannheim nor Popper take adequate account of the range of different types of scientific evidence that would be relevant to any social problem, and of the conflicts that can arise between them (Hammersley 2023a). They also both underemphasise the role that interests and ideology inevitably play in policymaking (Weiss 1983). Nor do they give sufficient attention to the essential role of experience‐based judgement on the part of policymakers, about what is and is not desirable and feasible, though this is perhaps less true of Mannheim than of Popper (Burke 1983; Hammersley 2024a).

## 6 Conclusion

In this article I have examined the views about social science and democratic governance put forward by two key figures in the history of political and social thought: Karl Mannheim and Karl Popper. They shared a technological conception of sociology and a commitment to democracy. However, there were deep differences in how they interpreted these ideas, signalled by Popper's severe criticisms of Mannheim. These differences illustrate the scope for variation *within* what Burawoy (2005) referred to as policy sociology, even aside from the contrasts between this and the other three sociological approaches he identified.

I examined the views of Mannheim and Popper about social science and its public role: one saw it as providing a comprehensive perspective on the character and trajectory of society that would serve as a basis for government planning, whereas the other viewed it as supplying specific information about particular social problems and how they could be remedied. I also considered these writers' conflicting views about the nature of democracy. Neither Popper nor Mannheim regarded democracy—in the sense of decision‐making by political representatives, elected by a widely‐defined citizenry, participating in a parliament, from whose members government ministers are appointed—as of value in itself. They saw it, rather, as a necessary means to serve other goals: in short, neither of them was concerned with sovereignty, but rather with good governance. I argued that, while Popper's fears about the dangers of Mannheim's democratic elitism carry some force, and Popper's position has much to commend it as an ideal, what he puts forward must be judged a utopia in light of the history of actually existing Western democracies. Mannheim is, perhaps, more realistic in recognising the need for a strong executive, not least to create and maintain the institutional arrangements in which rational public discourse can take place. This is also necessary, I suggested, if Popper's goal of reducing unnecessary suffering is to be achieved.

Mannheim assumes that sufficient political consensus can be established through education of the electorate, as a basis for substantial governmental planning. But, while he recognises the problem of ideological conflict, it is questionable whether his proposed solution is likely to be effective; and, as Popper indicates, it raises fears about indoctrination. By contrast, while Popper acknowledges the likelihood of significant differences in values and interests within a population, he seems to assume that these will not be a major obstacle in determining the nature of a social problem, deciding what potential solutions should be tried, assessing their effectiveness, and judging when sufficient improvement has been obtained. Here he underestimates the probability that there will be considerable public criticism of government and social science whatever policy is adopted, as well as the incapacity of many citizens properly to understand the social issues involved, and/or their unwillingness to make the effort to do this given other concerns.

Many have pointed to parallels between the situation in the West today and that of the 1930s, the period with which both Mannheim and Popper were preoccupied. One of these parallels is the problem of deep polarisations in political attitude within mass democracies. Another is the rise of populism, with some political leaders invoking the voice of the people against parliamentary representatives and the judiciary, and seeking to consolidate their own power in order to impose major policy changes (Hammersley 2023b). Both the authors I have discussed were aware of these problems, but they prioritised them differently. Mannheim believed that a strong executive could serve progressive common goals, whereas Popper was wary of the state even though he regarded it as necessary. Meanwhile, Mannheim feared the masses, in line with mass society theory (Yamada 2018), believing that control was required, notably via education, if potentially irrational demands were to be redirected. By contrast, Popper assumed the cognitive unity of humankind, insisting on people's capacity for rational thought and action, and viewed what Mannheim proposed as characteristic of totalitarianism. I have suggested that the views of both these authors are instructive, even if the solutions they offer are less than convincing.

Many of the issues raised here remain unresolved today (see, for instance, Wolf 2023).^27^ We are further away than in Mannheim's time from the sort of government planning to kerb the negative effects of capitalism that he recommended, but we are also more distant from the open society that Popper advocated. Finding ways to reconcile the freedom that is central to this type of society with the level of government intervention that Mannheim believed is required if what he regarded as civilised life is to be sustained remains a major challenge. Also unresolved is the question of what role sociology can play in this. But the work of these two thinkers can perhaps help us to understand these issues more clearly, and to recognise the range of alternative strategies available for dealing with them.

## Ethics Statement

The author has nothing to report.

## Consent

The author has nothing to report.

## Conflicts of Interest

The author declares no conflicts of interest.

## Permission to Reproduce From Material

The author has nothing to report.

## Data Availability

The author has nothing to report.

## Endnotes

^1^
Despite their deep differences in view, I suspect that Popper would have agreed with this statement wholeheartedly.

^2^
For a useful history of ‘the policy sciences’ in Western societies in the middle of the twentieth century, see Wagner et al. 1991. Subsequent relevant developments would include the rise of the notion of ‘knowledge societies’ (Stehr 1994) and of the idea of evidence‐based policymaking (Greenhalgh and Russell 2009; Hammersley 2012).

^3^
There are some parallels with Janowitz's (1972) contrast between an ‘enlightenment’ and an ‘engineering’ mode of the relationship between sociology and policymaking. See also Bulmer 1982:ch2.

^4^
My account of Mannheim's views here can be no more than a broad overview, and I do not discuss his earlier work in any detail, including *Ideology and Utopia* (Mannheim 1936). For a chronological account of Mannheim's publications, see Wolff (1971:Intro). For a discussion that locates his work in the context of ‘Weimar sociology’, see Kettler and Loader 2013. For the early formation of his thought in Hungary, see Karácsonyi 2008. Reynolds (2023) has argued that Mannheim's early work offers a via media between the critical rationalism of Popper and Adorno's critical theory.

^5^
For biographical information on Mannheim, see Wolff 1971:Intro and Kettler et al. 1984:Intro and Ch1. On Popper, see Hacohen 2000.

^6^
On the movement advocating planning, see Hammersley 2021. However, there were important differences in the positions adopted. For example, Keynes' advocacy of state planning was rather limited, primarily entailing the use of fiscal and monetary policy to control business cycles, and thereby avoid or minimise the effects of recessions (though see Winch 1969:Appendix). Like some others, including Emil Lederer (Marschak et al. 1941) and Karl Polanyi (1944), Mannheim went well beyond this.

^7^
For criticism of Popper's position from different political directions, see Rule 1978:ch4 and Avery 2003. See also Freeman's (1975) argument that Popper adopts an a priori sociology at odds with his view of scientific method, assumes a Manichean and a populist political perspective, as well as advancing his own version of utopianism.

^8^
On Popper's concept of an open society, compared to that of others, see Hammersley 2024b.

^9^
Some commentators have pointed to major differences in character between Mannheim's early and later work, including between *Ideology and Utopia* and *Man and Society* (Kettler et al. 1990), and these are certainly present. However, there are also important continuities. And some of the differences stem from Mannheim's move from addressing German‐language audiences to English‐language audiences. On the reception of *Ideology and Utopia* in the United States, see Kettler and Meja 1994.

^10^
For a brief outline of this debate, see Hammersley 2018:32–6.

^11^
For discussions of the notion of situational logic that illustrate its openness to a variety of interpretations, see Lagueux 2006; Bruin 2006.

^12^
Interestingly, there is a parallel with one of these types of synthesis, ‘meta‐ethnography’: in *Ideology and Utopia* Mannheim (1936, 270–1) argues that synthesis involves translating one ideology into the terms of another, and meta‐ethnography applies a similar process to different theories. However, we should note that Mannheim did not succeed in producing an overarching theoretical framework himself: his books are collections of essays, and they are not entirely consistent. There are significant discrepancies between them—for example the two very different conceptions of ‘ideology’ to be found even within *Ideology and Utopia*.

^13^
For a discussion of some of the complexities at the interface between scientific knowledge and policymaking, see Hammersley 2023a.

^14^
In his response to criticism from Rush Rhees, Popper (2008:ch15) denies he is proposing that social science should be technological in character: he insists that there can be ‘theoretical social sciences’, and that while these can learn from engineering, as can ‘theoretical natural sciences’, they are not equivalent to engineering. However, this denial does not fit with much of his discussion (Shearmur 1996, 187 Note 26).

^15^
In some respects, Mannheim's position, at least in parts of *IU*, also seems to be close to action research. Drawing on a claim about dialectical unification of theory and practice characteristic of Marxism, he argues that theory is a product of reality; such theory leads to action; actions attempt to change reality; in the case of failure, revision of the theory results; if the actions are successful they give rise to a new reality and to a new theory; and so on (Mannheim 1936, 112–3). It is worth noting that Karl Korsch, a Marxist influenced by Mannheim's early mentor, György *Lukács*, was an important influence on Lewin's action research: see Adelman 1993.

^16^
Town 1973 provides an excellent account of this and other ‘dilemmas’ in action research. On the argument that these arise, in part, from a misguided attempt to blend different forms of rationality, see Hammersley 2004.

^17^
There is also a semantic problem with Popper's position: that, in terms of the standard meaning of ‘knowledge’, as ‘justified true belief’, it implies that there can be no scientific knowledge at all. However, he would probably have dismissed this criticism as the product of an essentialist approach to the meaning of words.

^18^
Similarly, neither policymakers nor the bulk of citizens are likely to be convinced of the valuable role that science can play in shaping practice by Popper's argument that scientific investigation starts from problems and results in the recognition of further (albeit ‘deeper’) problems (Popper 1994, 157–9). For an argument that Popper's position implies a classical liberal view of the state, rather than the social democratic one that he espoused in his early career, see Shearmur 1996.

^19^
Popper later acknowledged this: ‘And it is a fact that my social theory (which favours gradual and piecemeal social reform, reform controlled by a critical comparison between expected and achieved results) contrasts with my theory of method, which happens to be a theory of scientific and intellectual revolution’ (Popper 1994, 68).

^20^
If we were to define a flourishing public sphere as the central feature of democracy—what is often referred to as discursive democracy—then Popper's commitment would be immediate not just instrumental.

^21^
There is a question about whether what Mannheim had in mind was planning by a central government: his notion of *principia media* (Mannheim 1940, 177–90), which is mentioned by Popper (1960, 101), suggests that there could be several planning agencies. However, it is not clear how the sort of societal‐wide coordination on key matters that he believed was necessary could be achieved in this way. And it seems likely that, under the form of governance he recommended, decisions would increasingly be made by relying on the specialist expertise claimed by a relatively closed, central elite.

^22^
There is a contrast between Popper's view of democracy and that of Max Weber (Beetham 1974) and Joseph Schumpeter (Swedberg 1991), who saw political leaders playing a much more active role in generating and orchestrating public opinion, as regards what are the most pressing problems and what should be done about them. This is closer to the conception of government adopted by Mannheim; though the latter seems to view the task of identifying and finding solutions to problems in more technical terms than they did.

^23^
This has long been recognised, see, for instance, the debate between Dewey and Lippmann in the 1920s (Ryan 1995, 216–20).

^24^
See Grant's (1997) argument that both Machiavelli and Rousseau insisted that political society necessarily depends upon deception and hypocrisy.

^25^
Where Popper regards openness as always positive, Mannheim is more circumspect: ‘The detrimental consequences of publicity in an increasing number of spheres can be best observed in the fatal fact that, since the World War, external politics in a certain sense have become increasingly public, and in these spheres waves of mass sentiment have had to be reckoned with where previously internal discussions in diplomatic circles would have sufficed. This is naturally no justification at all for the earlier secrecy and intrigue of international diplomacy. We must see, however, that we only fall from the frying pan of secret diplomacy into the fire of a foreign policy increasingly at the mercy of public whim and fancy’ (Mannheim 1940, 111).

^26^
This is an argument, with evidence supplied, that has been used by those who challenge the benefits of democracy: see, for example, Brennan 2016.

^27^
They also go back beyond the time of Mannheim and Popper: many can be found, in a somewhat different form, in the dispute between Hamilton and Madison in the early years of the United States: see Sheehan 2004.

## References

[bjos13221-bib-0001] Adelman, C. 1993. “Kurt Lewin and the Origins of Action Research.” Educational Action Research 1: 7–24. 10.1080/0965079930010102.

[bjos13221-bib-0002] Avery, T. 2003. “Popper on ‘Social Engineering’: A Classical Liberal View.” Reason Papers 26. https://www.reasonpapers.com/pdf/26/rp_26_3.pdf.

[bjos13221-bib-0003] Beetham, D. 1974. Max Weber and the Theory of Modern Politics. Allen & Unwin.

[bjos13221-bib-0004] Brennan, J. 2016. Against Democracy. Princeton University Press.

[bjos13221-bib-0005] Bruin, B. de. 2006. “Popper’s Conception of the Rationality Principle in the Social Sciences.” In Karl Popper: A Centenary Assessment, edited by I. Jarvie , K. Milford , and D. Miller , III. Ashgate.

[bjos13221-bib-0006] Buckley, W. 1967. Sociology and Modern Systems Theory. Prentice‐Hall.

[bjos13221-bib-0007] Bulmer, M. 1982. The Uses of Social Research. George Allen and Unwin.

[bjos13221-bib-0008] Burawoy, M. 2005. “For Public Sociology.” British Journal of Sociology 56, no. 2: 259–294: (Also published as: Burawoy, M. (2005). “For Public Sociology.” American Sociological Review 70, no. 1: 4–28). 10.1111/j.1468-4446.2005.00059.x. 15926908

[bjos13221-bib-0009] Burke, T. E. 1983. The Philosophy of Popper. Manchester University Press.

[bjos13221-bib-0010] Campbell, D. T. 1991. “Methods for the Experimenting Society.” Evaluation Practice 12, no. 3: 223–260. 10.1177/109821409101200304.

[bjos13221-bib-0011] Cohen, G. A. 1978. Karl Marx’s Theory of History: A Defence. Princeton University Press.

[bjos13221-bib-0013] Deaton, A. , and N. Cartwright . 2018. “Understanding and Misunderstanding Randomized Controlled Trials.” Social Science & Medicine 210: 2–21. 10.1016/j.socscimed.2017.12.005. 29331519 PMC6019115

[bjos13221-bib-0014] Floud, J. 1959. “Karl Mannheim.” In The Function of Teaching, edited by A. V. Judges . Faber and Faber.

[bjos13221-bib-0015] Freeman, M. 1975. “Sociology and Utopia: Some Reflections on the Social Philosophy of Karl Popper.” British Journal of Sociology 26, no. 1: 20–34. 10.2307/589240.

[bjos13221-bib-0016] Giddens, A. 1977. “Functionalism: Après la lutte.” In Studies in Social and Political Theory, edited by A. Giddens . Hutchinson.

[bjos13221-bib-0017] Grant, R. W. 1997. Hypocrite and Integrity: Machiavelli, Rousseau and the Ethics of Politics. University of Chicago Press.

[bjos13221-bib-0018] Greenhalgh, T. , and J. Russell . 2009. “Evidence‐based Policymaking: A Critique.” Perspectives in Biology and Medicine 52, no. 2: 304–318. 10.1353/pbm.0.0085. 19395827

[bjos13221-bib-0019] Gutting, G. 1999. Pragmatic Liberalism and the Critique of Modernity. Cambridge University Press.

[bjos13221-bib-0020] Hacohen, M. H. 2000. Karl Popper: The Formative Years 1902‐1945. Cambridge University Press.

[bjos13221-bib-0021] Hammersley, M. 2000. Taking Sides in Social Research. Routledge.

[bjos13221-bib-0022] Hammersley, M. 2004. “Action Research: A Contradiction in Terms?” Oxford Review of Education 30, no. 2: 165–181. 10.1080/0305498042000215502.

[bjos13221-bib-0023] Hammersley, M. 2013. The Myth of Research‐Based Policy and Practice. Sage.

[bjos13221-bib-0024] Hammersley, M. (2015) “The Mis‐Selling of Social Science”, Unpublished Paper: the‐misselling‐of‐social‐sciencefinal‐2‐3.doc (live.com)

[bjos13221-bib-0025] Hammersley, M. 2018. The Radicalism of Ethnomethodology. Manchester University Press.

[bjos13221-bib-0026] Hammersley, M. 2021. “Planning Versus the Market: The Dispute Between Hayek and Mannheim and Its Contemporary Relevance.” British Journal of Sociology 72, no. 5: 1464–1478. 10.1111/1468-4446.12893. 34519353

[bjos13221-bib-0027] Hammersley, M. (2022a) “Research‐based Policymaking and Practice: Science and Democracy in Harmony or Conflict?” ebp‐science‐and‐democracy‐28‐3.pdf (wordpress.com)

[bjos13221-bib-0028] Hammersley, M. 2022b. “Karl Mannheim’s *Ideology and Utopia* and the Public Role of Sociology.” Journal of Classical Sociology 22, no. 2: 176–198. 10.1177/1468795x20986382.

[bjos13221-bib-0029] Hammersley, M. 2023a. “‘Was the UK Government’s Policymaking ‘Evidence‐Based’ During the Pandemic? Reflections on Science and Politics.” Zeitschrift für Evaluation 2023, no. 2: 217–242. 10.31244/zfe.2023.02.03.

[bjos13221-bib-0030] Hammersley, M. 2023b. “Karl Mannheim on Fascism: Sociological Lessons About Populism and Democracy Today?” Sociological Research Online 28, no. 2: 320–335. 10.1177/13607804211042032.

[bjos13221-bib-0031] Hammersley, M. 2024a. “Challenging Historicist Utopianism: Karl Popper on Karl Mannheim.” History of European Ideas: 1–19. 10.1080/01916599.2024.2437376.

[bjos13221-bib-0032] Hammersley, M. 2024b. “What Is an ‘Open Society’? Bergson, Strauss, Popper, and Deleuze.” History of European Ideas 50, no. 8: 1422–1432. 10.1080/01916599.2024.2365143.

[bjos13221-bib-0033] Hayek, F. 1943. “The Facts of the Social Sciences.” Ethics 54, no. 1: 1–13. 10.1086/290368.

[bjos13221-bib-0034] Janowitz, M. 1972. Sociological Models and Social Policy, General Learning systems.

[bjos13221-bib-0035] Karácsonyi, A. 2008. “Soul‐Life‐Knowledge: The Young Mannheim’s Way to Sociology.” Studies in East European Thought 60, no. 1–2: 97–111. 10.1007/s11212-008-9040-4.

[bjos13221-bib-0036] Kettler, D. 1975. “Political Theory, Ideology, Sociology: The Question of Karl Mannheim.” Cultural Hermeneutics 3, no. 1: 69–80. 10.1177/019145377500300107.

[bjos13221-bib-0037] Kettler, D. , and C. Loader . 2013. “Weimar Sociology.” In Weimar Thought : A Contested Legacy, edited by P. E. Gordon and J. P. McCormick , Princeton University Press.

[bjos13221-bib-0038] Kettler, D. , and V. Meja . 1994. “‘That Typically German Kind of Sociology Which Verges Towards Philosophy’: The Dispute About *Ideology and Utopia* in the United States.” Sociological Theory 12, no. 3: 279–303. 10.2307/202126.

[bjos13221-bib-0039] Kettler, D. , V. Meja , and N. Stehr . 1984. Karl Mannheim. Tavistock.

[bjos13221-bib-0040] Kettler, D. , V. Meja , and N. Stehr . 1990. “Rationalizing the Irrational: Karl Mannheim and the Besetting Sin of German Intellectuals.” American Journal of Sociology 95, no. 6: 1441–1473. 10.1086/229460.

[bjos13221-bib-0041] Lagueux, M. 2006. “Popper and the Rationality Principle.” In Karl Popper: A Centenary Assessment, edited by I. Jarvie , K. Milford , and D. Miller , Vol. III. Ashgate.

[bjos13221-bib-0042] Lewin, K. 1946. “Action Research and Minority Problems.” Journal of Social Issues 2, no. 4: 34–46: [Reprinted in K. Lewin (1977) Resolving Social Conflicts and Field Theory in Social Science, Washington DC, American Psychological Association.]. 10.1111/j.1540-4560.1946.tb02295.x.

[bjos13221-bib-0043] Loader, C. 1985. The Intellectual Development of Karl Mannheim. Cambridge University Press.

[bjos13221-bib-0044] Luhmann, N. 2012. Theory of Society, 1. Stanford University Press.

[bjos13221-bib-0045] Mannheim, K. 1936. Ideology and Utopia. Routledge and Kegan Paul: First published in German in 1929.

[bjos13221-bib-0046] Mannheim, K. 1940. Man and Society in an Age of Reconstruction. Kegan Paul, Trench, Trubner and Co: First published in German in 1936.

[bjos13221-bib-0047] Mannheim, K. 1953. “Conservative Thought.” In Essays in Sociology and Social Psychology, edited by K. Mannheim Routledge and Kegan Paul: First published in German in 1927.

[bjos13221-bib-0048] Marrow, A. 1969. The Practical Theorist: The Life and Work of Kurt Lewin. Teachers College Press.

[bjos13221-bib-0049] Marschak, J. , A. Kähler , and E. Heiman . 1941. “Emil Lederer, 1882–1939: II. The Economist.” Social Research 8, no. 1: 79–105.

[bjos13221-bib-0050] Polanyi, K. 1944. The Great Transformation. Farrar and Rinehart.

[bjos13221-bib-0051] Popper, K. R. 1944–1945. “The Poverty of Historicism I, II, III.” Economica 11, no. 42: 86–103: 11, 43, 119–37;12, 46, 69–89. 10.2307/2549642.

[bjos13221-bib-0052] Popper, K. R. 1945a. The Open Society and Its Enemies, 1. Routledge and Kegan Paul. 10.4324/9780203538111.

[bjos13221-bib-0053] Popper, K. R. 1945b. The Open Society and Its Enemies, 2. Routledge and Kegan Paul. 10.4324/9780203538111.

[bjos13221-bib-0054] Popper, K. R. 1960. The Poverty of Historicism. 2nd ed. Routledge and Kegan Paul.

[bjos13221-bib-0056] Popper, K. R. 1966. The Open Society and Its Enemies. 2. Revised edition Routledge.

[bjos13221-bib-0057] Popper, K. R. 1994. The Myth of the Framework. Routledge.

[bjos13221-bib-0058] Popper, K. R. 2008. After the Open Society: Selected Social and Political Writings. edited by J. Shearmur and P. N. Turner , Routledge.

[bjos13221-bib-0059] Rescher, N. 1978. Peirce’s Philosophy of Science. Notre Dame University Press.

[bjos13221-bib-0060] Reynolds, I. 2023. Education for Political Life: Critique, Theory, and Practice in Karl Mannheim's Sociology of Knowledge. Rowman and Littlefield.

[bjos13221-bib-0061] Rule, J. B. 1978. Insight and Social Betterment. Oxford University Press.

[bjos13221-bib-0062] Ryan, A. 1995. John Dewey and the High Tide of American Liberalism. W. W. Norton.

[bjos13221-bib-0063] Shearmur, J. 1996. The Political Thought of Karl Popper. Routledge.

[bjos13221-bib-0064] Sheehan, C. A. 2004. “Madison V. Hamilton: The Battle over Republicanism and the Role of Public Opinion.” American Political Science Review 98, no. 3: 405–424. 10.1017/s0003055404001248.

[bjos13221-bib-0065] Smith, C. 2014. The Sacred Project of American Sociology. Oxford University Press.

[bjos13221-bib-0066] Stehr, N. 1994. Knowledge Societies. Sage.

[bjos13221-bib-0067] Stokes, G. 1998. Popper: Philosophy, Politics and Scientific Method. Polity.

[bjos13221-bib-0068] Swedberg, R. 1991. Joseph A. Schumpeter: His Life and Work. Polity Press.

[bjos13221-bib-0069] Town, S. 1973. “Action Research and Social Policy: Some Recent British Experience.” Sociological Review 21, no. 4: 573–598. 10.1111/j.1467-954x.1973.tb00498.x.

[bjos13221-bib-0070] Turner, S. P. 1992. “Sociology and Fascism in the Interwar Period: The Myth and its Frame.” In Sociology Responds to Fascism, edited by S. P. Turner and D. Käsler , 1–13. Routledge.

[bjos13221-bib-0071] Turner, S. P. 2014. The Politics of Expertise. Routledge.

[bjos13221-bib-0072] Van Parijs, P. 1981. Evolutionary Explanation in the Social Sciences. Tavistock.

[bjos13221-bib-0073] Wagner, P. , C. H. Weiss , B. Wittrock , and H. Wollman , eds. 1991. Social Sciences and Modern States. Cambridge University Press.

[bjos13221-bib-0074] Weiss, C. 1983. “Ideology, Interests, and Information.” In Ethics, the Social Sciences, and Policy Analysis, edited by D. Callahan and B. Jennings , Plenum Press.

[bjos13221-bib-0075] Winch, D. 1969. Economics and Policy: A Historical Study. Hodder and Stoughton.

[bjos13221-bib-0076] Wolf, M. 2023. The Crisis of Democratic Capitalism. Allen Lane.

[bjos13221-bib-0077] Wolff, K. H. ed. 1971. From Karl Mannheim. Oxford University Press

[bjos13221-bib-0078] Yamada, R. 2018. “Mannheim, Mass Society, and Democratic Theory.” In The Anthem Companion to Karl Mannheim, edited by D. Kettler and V. Meja , Anthem Press.

